# What are the main proteins in the hemolymph *of Haemaphysalis flava* ticks?

**DOI:** 10.3389/fvets.2024.1387719

**Published:** 2024-07-17

**Authors:** Dan Li, Lei Liu, Zi-ling Liu, Yuan Tian, Xin Gao, Tian-yin Cheng

**Affiliations:** Research Center for Parasites and Vectors, College of Veterinary Medicine, Hunan Agricultural University, Changsha, Hunan, China

**Keywords:** *Haemaphysalis flava*, hemolymph, vitellogenin, carrier protein, α-macroglobulin

## Abstract

**Background:**

*Haemaphysalis flava* is a notorious parasite for humans and animals worldwide. The organs of *H. flava* are bathed in hemolymph, which is a freely circulating fluid. Nutrients, immune factors, and waste can be transported to any part of the body via hemolymph. The main soluble components in hemolymph are proteins. However, knowledge of the *H. flava* proteome is limited.

**Methods:**

The hemolymph was collected from fully engorged *H. flava* ticks by leg amputation. Hemolymph proteins were examined by both blue native polyacrylamide gel electrophoresis (BN-PAGE) and sodium dodecyl sulfate PAGE (SDS-PAGE). Proteins extracted from the gels were further identified by liquid chromatography–tandem mass spectrometry (LC–MS/MS).

**Results:**

Two bands (380 and 520 kDa) were separated from tick hemolymph by BN-PAGE and were further separated into four bands (105, 120, 130, and 360 kDa) by SDS-PAGE. LC–MS/MS revealed that seven tick proteins and 13 host proteins were present in the four bands. These tick proteins mainly belonged to the vitellogenin (Vg) family and the α-macroglobulin family members. *In silico* structural analysis showed that these Vg family members all had common conserved domains, including the N-terminus lipid binding domain (LPD-N), the C-terminus von Willebrand type D domain (vWD), and the domain of unknown function (DUF). Additionally, two of the Vg family proteins were determined to belong to the carrier protein (CP) by analyzing the unique N-terminal amino acid sequences and the cleaving sites.

**Conclusion:**

These findings suggest that the Vg family proteins and α-macroglobulin are the primary constituents of the hemolymph in the form of protein complexes. Our results provide a valuable resource for further functional investigations of *H. flava* hemolymph effectors and may be useful in tick management.

## 1 Introduction

Diseases caused by *Haemaphysalis flava* can influence human and animal health and have a major economic impact. *H. flava* is commonly found in the Asian continent, including China, South Korea, Vietnam, and Japan (1). *H. flava* can infect a wide range of animals, including humans, domestic animals (dogs, pigs, horses, sheep, and cattle), and wildlife (hedgehogs, water deer, Raccoon dogs, eastern roe deer, and pandas) (2–4). *H. flava* plays the role of a vector in the transmission process of bacterial pathogens, parasites, and viruses in these animals (5–7).

Hemolymph is an important exchange and transport medium in ticks. It is a circulating fluid that bathes all internal tissues and organs in ticks, similar to the blood and lymph in vertebrates. It is composed of hemocytes and plasma (8). The plasma contains proteins, lipids, carbohydrates, and hormones and is involved in the transport of nutrients, metabolites, hormones, and even specific pathogenic microorganisms (9). Proteins are the main soluble component in the hemolymph and are crucial in many physiological processes, such as the regulation of osmotic pressure and innate immunity (10, 11). However, studies on the main proteins in tick hemolymph remain relatively limited.

Proteomics has continued to be the preferred method for studying tick proteins in recent years. Numerous researchers have attempted to identify and isolate hemolymph proteins in ticks. At the very beginning, Belozerov and Luzev separated 25 protein bands from the hemolymph of the tick *Dermacentor marginatus Sulz* using polyacrylamide gel electrophoresis (12). At present, the proteins studied exclusively are two storage proteins, namely, carrier protein (CP) and vitellogenin (Vg). Because of the similarities between CP and Vg, they have been thought to have a common evolutionary origin (13, 14). In our study, CP and Vg are collectively referred to as the Vg family. The role of Vg in ticks is to supply nutrients to growing embryos as a yolk protein precursor (15). CP is involved in the storage and transportation of carbohydrates and lipids such as free fatty acids, free cholesterol, and monoacylglycerol (16). In addition to Vg and CP, many proteins, such as α-macroglobulins, lectins, and antimicrobial peptides, are associated with tick immunity and are secreted into the hemolymph in response to injury and pathogen invasion (17–19). In spite of the importance of these proteins, our knowledge about them is limited relative to ticks.

Previously, we provided an overview of the components in the hemolymph of *H. flava* (8). There are 312 proteins, including tick-derived proteins and host-derived proteins. Unlike previous studies, we further separated the biomacromolecules of hemolymph mainly by blue native polyacrylamide gel electrophoresis (BN-PAGE) and sodium dodecyl sulfate PAGE (SDS-PAGE). Furthermore, the composition of the biomacromolecules was determined and analyzed by LC–MS/MS. These findings add to our understanding of the vital role of these proteins in the physiology of the tick circulatory system and provide additional potential targets for the development of tick control methods.

## 2 Materials and methods

All procedures performed in this study involving animals were in accordance with the ethical standards of the Hunan Agricultural University Institutional Animal Care and Use Committee (No. 2021085).

### 2.1 Tick hemolymph collection

Ticks were collected and identified on the basis of morphology and molecular biology, as previously described (20). The fully engorged females (300–350 mg in weight) were picked from hedgehogs in Xinyang City, Henan Province, China (31°44′N, 114°10′E).

After being rinsed three times with distilled water and sterilized with 70% ethanol, 50 ticks were immobilized on a sterile glass slide with tape. The femurs were cut off, and the hemolymph was collected with a pipette pre-filled with a protease inhibitor cocktail (Phygene, Fujian, China). The mixture was centrifuged at 14,000 *g* for 10 min at 4°C, and the supernatant was stored at 80°C for subsequent analysis.

### 2.2 Electrophoresis analysis of hemolymph proteins

Protein concentrations were measured using a BCA protein assay kit (Applygen, Beijing, China). For BN-PAGE, the sample preparation and procedures were carried out according to the Blue/Clear Native PAGE Electrophoresis Kit (Real Times, Beijing, China). Briefly, 6.5 μL samples (6.04 μg/μL) were mixed with 2.5 μL of 4 × BN/CN-PAGE protein loading buffer (Real Times, Beijing, China) and 1 μL of 5% G-250 dyestuff (Real Times, Beijing, China). After mixing, each sample was subjected to BN-PAGE (4–16%, Real Times, Beijing, China) at 4°C at 5–15 V for 3 h. After electrophoresis, proteins in the gels were stained with Coomassie brilliant blue and scanned with a ScanMaker i800 plus (Microtek, Shanghai, China). For SDS-PAGE, BN-PAGE lanes were cut out of the gels with a razor blade, transferred to sterile microtubes, and incubated in the SDS protein loading buffer (TransGen, Beijing, China). They were ground and centrifuged at 14,000 × *g* at 4°C for 10 min. The mixture was subjected to a boiling-water bath for 5 min. After being cooled and centrifuged at 14,000 × g for 10 min at 4°C, samples were loaded into the wells of precast gels (4–12%, Epizyme, Shanghai, China). Electrophoresis was conducted at 150 V for 1 h. After electrophoresis, gels were stained with Coomassie brilliant blue for proteins. The target bands were cut, and the proteins were digested in the gels and analyzed by LC–MS/MS.

### 2.3 Digestion of the proteins in the gels

The clear and highly dyed protein bands were cut from the gels and put into tubes. Then, acrylonitrile (ACN) was added to decolorize the gels, which were cleaned with ultrapure water until transparent. After adding 10 μL of 100 mM dithiothreitol (DTT) and 90 μL of 100 mM ammonium bicarbonate (NH_4_HCO_3_) to each tube, the liquid was incubated at 56°C for 30 min. Then, it was set in a dark room for 30 min with 30 μL of 200 mM iodoacetamide (IAA), 70 μL of 100 mM NH_4_HCO_3_, and further rinsed with 100 mM of NH_4_HCO_3_ and ACN. The mixture was incubated overnight at 37°C after adding 12.5 ng/mL of trypsin and 25 mM of NH_4_HCO_3_ mixture. The peptides were extracted with 60% ACN and 0.1% trifluoroacetic acid (TFA) three times. The extracts were pooled and dried completely by vacuum centrifuge (21).

### 2.4 Analysis by LC–MS/MS

MS analyses were carried out on a Q Exactive mass spectrometer coupled to Easy nLC (Thermo Fisher Scientific, Waltham, MA, United States) following the same conditions previously described (8). MS data were obtained using data-dependent top 10 methods, which dynamically selected the most abundant precursor ions from the survey scans (300–1800 m/z) for higher-energy collisional dissociation (HCD) fragmentation. The target value was determined based on predictive automatic gain control. The dynamic exclusion duration was set to 25 s. The resolution for survey scans and HCD spectra was set to 70,000 at 200 m/z and 17,500 at 200 m/z, respectively. The normalized collision energy was set to 30 eV. The fill ratio was defined as 0.1%.

### 2.5 Sequence database searches and data processing

MS data were handled using MaxQuant software (version 1.6.14.0).^1^ The MS/MS raw files were searched against the self-constructed *H. flava* protein database deduced from transcriptomic data^2^^,^^3^^,^^4^^,^^5^ for identifications of tick proteins. An initial search was set with a precursor quality window of 6 ppm. The search followed the enzymatic cleavage rule of trypsin/P and allowed a maximum of two missed cleavage sites and a mass tolerance of 20 ppm for fragment ions. Carbamidomethylation of cysteines was defined as a fixed modification, while protein N-terminal acetylation and methionine oxidation were defined as variable modifications. The cutoff of the global false discovery rate for peptide and protein identification was set at 0.01. Protein abundance was calculated based on the normalized spectral protein intensity. The sequence analysis was conducted using DNAMAN and IBS.^6^

## 3 Results

**Figure 1:**
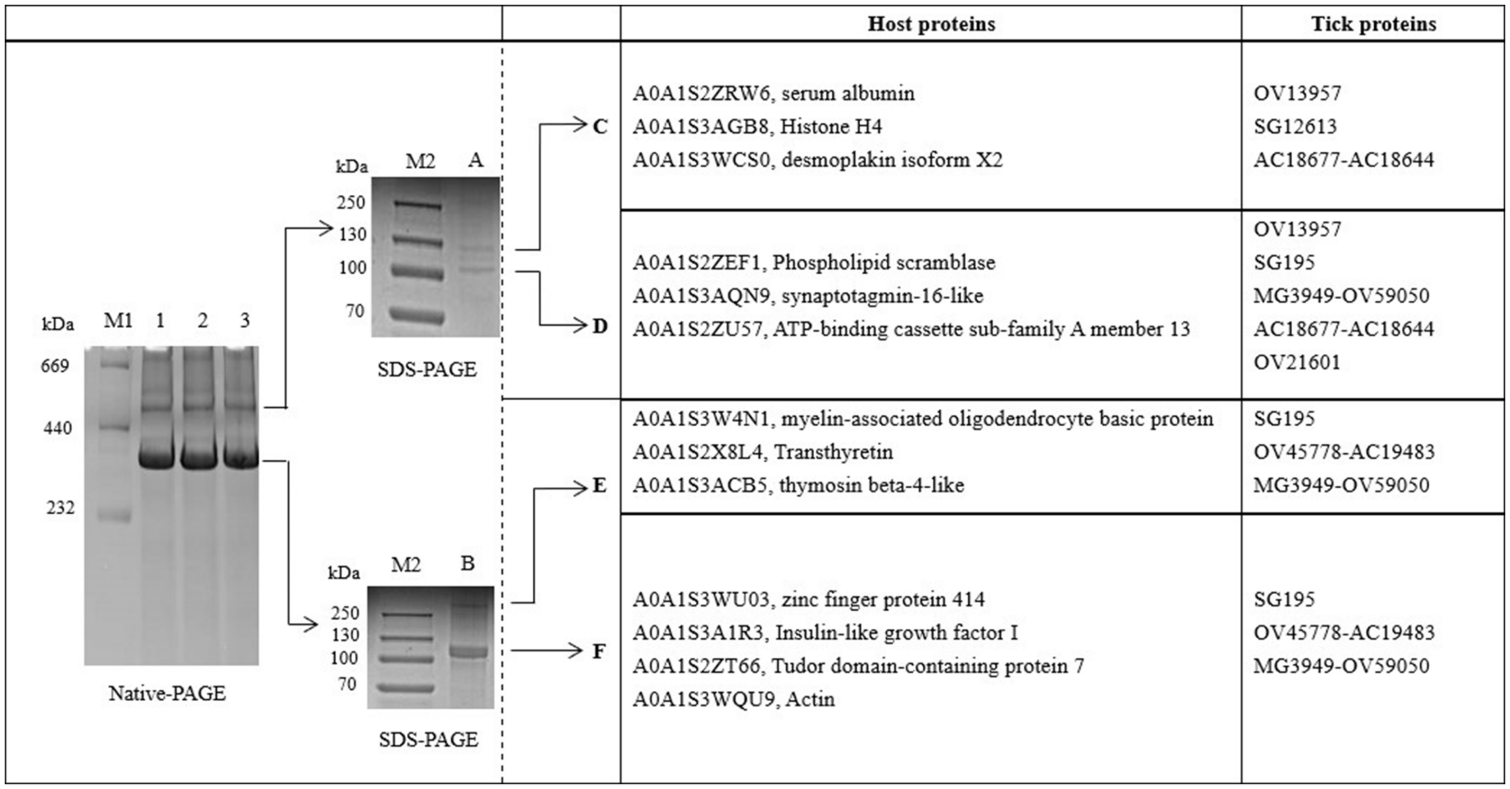
BN-PAGE and SDS-PAGE analysis of hemolymph proteins in *Haemaphysalis flava*. OV13957, SG195, OV45778-AC19483, MG3949-OV59050, OV21601, and AC18677-AC18644 are protein IDs in the self-constructed *H. flava* protein database. They can be annotated as Vg-1, Vg-1, Vg-2, Vg-2, Vg-2, α-macroglobulin, and α-1-macroglobulin.

The proteins in *H. flava* hemolymph were measured by a BCA assay. The protein concentration of tick hemolymph was 6.04 μg/μL. To examine the main proteins of the hemolymph in more detail, we used BN-PAGE to separate protein complexes by their size. The result showed that there were two bands with proteins isolated from the hemolymph; we called them A and B. The native protein complexes were found to have molecular weights of 520 and 380 kDa. SDS-PAGE, followed by BN-PAGE, indicated an enrichment of specific proteins. The protein complex A (520 kDa) was separated into band C (130 kDa) and D (105 kDa), while the protein complex B (380 kDa) was separated into band E (360 kDa) and F (120 kDa) (see Figure 1).

Bands C, D, E, and F were sliced, digested with trypsin, and analyzed by LC–MS/MS. Protein annotation using UniProt confirmed the presence of seven tick-derived proteins and 13 host proteins. These tick-derived proteins mainly belong to the Vg family and the α-macroglobulin family (see Table 1). The host protein types were as follows: histone H4, desmoplakin isoform X2, serum albumin, ATP-binding cassette sub-family A member 1, actin, phospholipid scramblase, synaptotagmin-16-like, myelin-associated oligodendrocyte basic protein, transthyretin, thymosin beta-4-like, zinc finger protein 414, insulin-like growth factor I, and Tudor domain-containing protein 7 (see Table 2).

**Table 1:** High confidence tick proteins in the hemolymph of *Haemaphysalis flava* ticks.

Polypeptide ID	UniProt accession	Protein name	Organism	*E*-value	Score	Identity (%)
OV13957	A0A346JM05	Vitellogenin-1	*Haemaphysalis flava*	0	8,535	92.9
SG12613	A0A346JM05	Vitellogenin-1	*H. flava*	0	8,294	100
SG195	A0A346JM06	Vitellogenin-2	*H. flava*	0	5,619	100
OV45778-AC19483	A0A346JM06	Vitellogenin-2	*H. flava*	0	5,408	64.3
MG3949-OV59050	A0A346JM06	Vitellogenin-2	*H. flava*	0	2,524	85.2
OV21601	A0A1E1X3E1	Alpha-macroglobulin	*Amblyomma aureolatum*	0	4,969	81.4
AC18677-AC18644	A0A023FY55	Alpha-1-macroglobulin	*Amblyomma parvum*	0	7,027	89.1

**Table 2:** High confidence host (*Erinaceus europaeus*) proteins in the hemolymph of *Haemaphysalis flava* ticks.

Protein name	UniProt Accession	Organism	No. of unique peptides	Coverage (%)
Histone H4	A0A1S3AGB8	*Erinaceus europaeus*	4	42.7
Desmoplakin isoform X2	A0A1S3WCS0	*Erinaceus europaeus*	3	1.3
Serum albumin	A0A1S2ZRW6	*Erinaceus europaeus*	3	4.3
ATP-binding cassette sub-family A member 1	A0A1S2ZU57	*Erinaceus europaeus*	3	0.3
Actin	A0A1S3WQU9	*Erinaceus europaeus*	3	9.3
Phospholipid scramblase	A0A1S2ZEF1	*Erinaceus europaeus*	1	4.5
Synaptotagmin-16-like	A0A1S3AQN9	*Erinaceus europaeus*	1	2.3
Myelin-associated oligodendrocyte basic protein	A0A1S3W4N1	*Erinaceus europaeus*	1	7.3
Transthyretin	A0A1S2X8L4	*Erinaceus europaeus*	1	9
Thymosin beta-4-like	A0A1S3ACB5	*Erinaceus europaeus*	1	25
Zinc finger protein 414	A0A1S3WU03	*Erinaceus europaeus*	1	2.1
Insulin-like growth factor I	A0A1S3A1R3	*Erinaceus europaeus*	1	7.8
Tudor domain-containing protein 7	A0A1S2ZT66	*Erinaceus europaeus*	1	1.1

**Figure 2:**
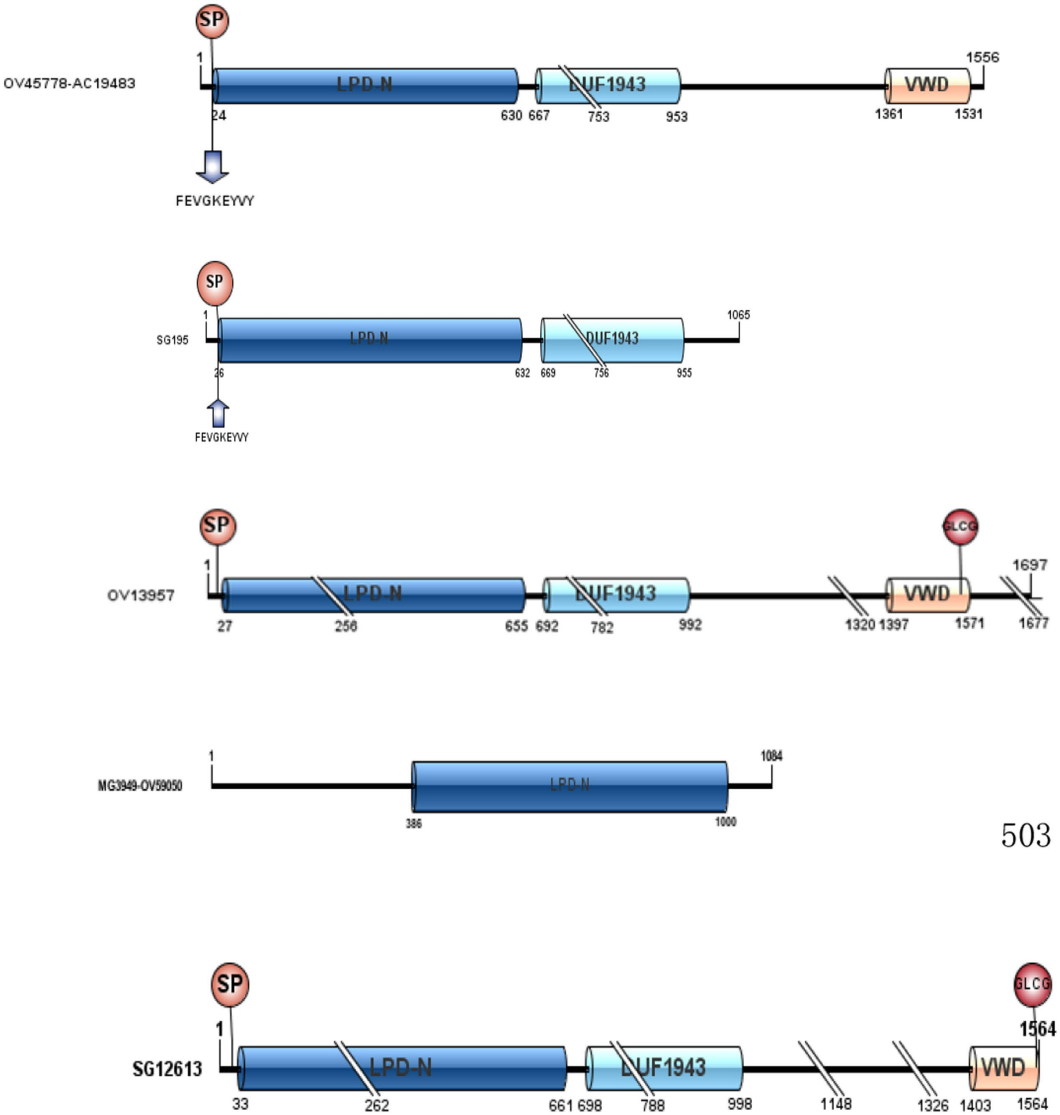
LPD_N, DUF1943, and vWD of the five Vg family proteins in *Haemaphysalis flava* hemolymph.

To better understand the function of the Vg family proteins, we analyzed the structure of Vg identified in our study using DNAMAN and the IBS software analysis tools. The Vg family proteins have three conserved regions, namely, the N-terminal lipid binding domain (LPD_N), the C-terminal von Willebrand type D domain (vWD), and the domain of unknown function (DUF) (see Figure 2). In addition, the RXXR highly conserved unique N-terminal amino acid sequences (FEVGKEYVY), and GLCC are partially present in the Vg family of proteins.

## 4 Discussion

### 4.1 Overview of the hemolymph protein complexes of *Haemaphysalis flava*

The biomacromolecules in *H. flava* by Native PAGE have not been reported, but they have been reported in *Dermacentor* var*iabilis* and *Ornithodoros parkeri* (22). Thompson et al. found a Vg protein with a molecular weight between 198 and 443 kDa by Native PAGE in pre-ovipositing and ovipositing replete (mated) females (15). Gudderra et al. purified and characterized a novel lipoglycoheme-carrier protein (CP) in *D. variabilis* by Native PAGE from partially fed virgin female hemolymph. The CP has a molecular weight of 200 kDa by Native PAGE and 340 kDa by gel filtration chromatography (16). Meanwhile, a high molecular weight protein (500 K) was found in *O. parkeri* (15, 16). These studies agree that Vg and CP are the main proteins in tick hemolymph. In addition, the three most abundant host-derived proteins in the hemolymph of *H. flava* are hemoglobin subunit-α, subunit-β, and albumin and the tick-derived proteins are Vg, microplusin, and α-2macroglobulin (8). Consistent with these studies, our study also found two high molecular weight proteins (520 kDa/380 kDa), and their main components were identified as Vg, α-macroglobulin, serum albumin, etc.

### 4.2 Vg family proteins

Protein domains are substructures of proteins that may give clues to their functional analysis. Therefore, we analyzed the structure of the Vg family of proteins characterized in the current study. Previous studies have shown that Vgs are proteins of 400–600 kDa and exist in oligomeric forms in the insect hemolymph (15, 23, 24). However, in *H. flava*, no such similar rule was observed in terms of the molecular weight and the subunit number of Vg. In the current study, three conserved regions (LPD_N, vWD, and DUF) exist in the Vg family of proteins. LPD-N is the most significant phosphorylation site of Vg and also an important protein modification region. It plays an important role in Vg splicing, Vg-VgRs recognition, and its mediated nutrient transport. vWD and DUF interact with pathogenic microorganisms, such as viruses or bacteria, which in turn eliminates pathogenic microorganisms (25). In 1988, Baker et al. noticed that the vWD domain might be involved in binding the Vg receptor present on the surface of oocytes by drawing comparisons between the vWD domain of Vgs and the vW factor (26). Interestingly, these structures can be found in comparable locations to DvCP, AaCP, and HlVg-C (14). In addition, we found that RXXR exists in these proteins. The RXXR can be recognized by subtilisin-like proprotein convertases, and the protein can be cleaved into multiple subunits. Thompson et al. showed that DvVg has an RXXR and would be split into two subunits (15). Therefore, this may be related to the fact that Vg appeared in different bands in our study. Meanwhile, SG195 and OV45778-AC19483 were discovered to have highly conserved unique N-terminal amino acid sequences (FEVGKEYVY) and a single cleavage site (RXXR)., which could be called CP (23). Khail et al. studied CP and Vg in detail. They reported that Vgs usually have many subunits, whereas CPs only have two subunits. In other words, the CP usually has one RXXR, and Vg has multiple RXXRs. SG12613 and OV13957 contain one to several cleavage sites and have non-conservative N-terminal amino acid sequences (Figure 2), which could be called Vg. This is consistent with our previous research. In our laboratory, we found that four proteins (Cl-33,847.0, Cl-k.7718, Cl-k.65658, and Cl-k.17389) were consumed in *H. flava* eggs, and we speculated that these proteins supported the embryonic development as precursors of Vn (27). SG195 and OV45778-AC19483 were not present in these proteins, which meant they were not Vg but CP.

Both Vg and CP belong to the Vg family. Vg is known to be a yolk protein precursor supplying nutrients to growing embryos (28). CP is thought to be a transporter of carbohydrates and lipids (16). Vg is mainly synthesized by the fat body and gut cells, then expelled into the hemolymph and finally taken up by the oocytes (29). Similar to Vg, CP is synthesized in the fat body and found in the midgut, salivary glands, hemolymph, and ovary (13, 22, 30–34). In our study, Vg and CP exist in the hemolymph as a protein complex. Some studies suggest that the abundance of CP was affected by blood feeding in females. Compared to unfed adult females, the abundance of CP declined in partially fed females and replete females. In addition, CP was found in 1- and 9-day-old eggs (35). Therefore, CP may act as a carrier for Vg to transport it to the egg to play a role.

### 4.3 Tick α-macroglobulin

An essential component, α-macroglobulin, was identified in the electrophoretic strip. TAM is considered to be a universal protease inhibitor, including serine-, cysteine-, aspartic-, and metalloproteases, on which the digestion of blood meal depends (36). In invertebrates, our knowledge of the α-macroglobulin in ticks is minimal. The α2M family of protease inhibitors so far are single chains with a molecular mass of approximately 180 kDa, and they can form tetramers or dimers or remain as monomers (17). The structure is similar to that of αM purified from the soft tick (17); α-macroglobulin in our study was a protein of approximately 520 kDa in the native state and dissociated on reducing SDS-polyacrylamide gels into two subunits with a mass of 130 and 105 kDa. Kopáček also reported that TAM inhibits trypsin and thermolysin cleavage of the high-molecular-weight substrate azocoll in a manner similar to that of bovine α-2-macroglobulin. In other studies, TAM protects multiple proteins from enzymatic hydrolysis (37) and is related to hormone transportation (38). Meanwhile, TAM is also a transmembrane molecule related to a low-density lipoprotein. Therefore, we speculate that TAM may be involved in lipoprotein transport with CP in tick hemolymph.

Recent discoveries have revealed hundreds of different proteins in tick hemolymph. The most prominent proteins are Vg, microplusin, macroglobulin, metalloproteinase, and serpins (8, 39). However, apart from Vg and macroglobulin, we did not detect any other proteins. Because these small molecular proteins are dispersed in bands and do not bind to larger proteins, such as Vg, these proteins cannot be seen and identified in electrophoretic bands.

The biomacromolecular in *H. flava* by Native PAGE has not been reported, but it has been reported in *Dermacentor* var*iabilis* and *Ornithodoros parkeri* (22). Thompson et al. found a Vg protein with a molecular weight between 198 and 443 kDa by Native PAGE in pre-ovipositing and ovipositing replete (mated) females (15). Gudderra et al. purified and characterized a novel lipoglycoheme-carrier protein (CP) in *D.* var*iabilis* by Native PAGE from partially fed virgin female hemolymph. The CP has a molecular weight of 200 kDa by Native PAGE and 340 kDa by gel filtration chromatography (16). Meanwhile, they found a high molecular weight protein (500 K) in *O. parkeri* (15, 16). These studies agree that Vg and CP are the main proteins in tick hemolymph. The top three abundant host-derived proteins in the hemolymph of *H. flava* are hemoglobin subunit-α, subunit-β, and albumin and tick-derived proteins are Vg, microplusin, and α-2macroglobulin (8). Consistent with these studies, our study also found two high molecular weight proteins (520 kDa/380 kDa), and their main component was identified as Vg, α-macroglobulin, serum albumin, etc.

The biomacromolecular in *H. flava* by Native PAGE has not been reported, but it has been reported in *Dermacentor variabilis* and *Ornithodoros parkeri* (22). Thompson et al. found a Vg protein with molecular weight between 198 and 443 kDa by Native PAGE in pre-ovipositing and ovipositing replete (mated) females (15). Gudderra et al. purified and characterized a novel lipoglycoheme-carrier protein (CP) in *D. variabilis* by Native PAGE from partially fed virgin female hemolymph. The CP has a molecular weight of 200 kDa by Native PAGE and 340 kDa by gel filtration chromatography (16). Meanwhile, they found a high molecular weight protein (500 K) in *O. parkeri* (15, 16). These studies agree that Vg and CP are the main proteins in tick hemolymph. The top three abundant host-derived proteins in the hemolymph of *H. flava* are hemoglobin subunit-α, subunit-β, and albumin and tick-derived proteins are Vg, microplusin, and α-2macroglobulin (8). Consistent with these studies, our study also found two high molecular weight proteins (520 kDa/380 kDa), and their main component was identified as Vg, α-macroglobulin, serum albumin, etc.

### 4.4 Host proteins

Ticks are obligatory blood-feeding ectoparasites that ingest a significant volume of host blood. Therefore, the host proteins are abundant in tick hemolymph. Liu et al. revealed that 40 host-derived proteins, such as hemoglobin subunit-α and subunit-β, albumin, serotransferrin-like, and ubiquitin-like, were present in the hemolymph of *H. flava*. It is worth mentioning that hemoglobin is the most abundant host protein; however, it was not found in our study. At present, we have found that the majority of the hemoglobin present in ticks is related to antimicrobial peptides (40, 41). Therefore, hemoglobin may degrade into small antimicrobial peptides without our detection. In our study, we found an important host protein, serum albumin, present in hemolymph at a higher percentage. Serum albumin is the main protein in the blood plasma and functions as a carrier, chaperone, antioxidant, source of amino acids, osmoregulatory, etc. (42). Serum albumin is also a fatty acid-binding protein. Fatty acids are one of the most important sources for maintaining metabolism and energy homeostasis in cells (43). Serum albumin possesses approximately 7 binding sites for fatty acids with moderate to high affinity (44). Hence, it can transport bulk amounts of FA into the hemolymph. Moreover, serum albumin plays a very important role in the uptake of FA by organs. This study could be useful in understanding the fatty acid metabolism and transport in ticks and presents a potentially major contribution to research on this molecule.

Finally, these bands were found to contain 12 other host proteins, namely, histone H4, desmoplakin isoform X2, ATP-binding cassette sub-family A member 1, actin, phospholipid scramblase, synaptotagmin-16-like, myelin-associated oligodendrocyte basic protein, transthyretin, thymosin beta-4-like, zinc finger protein 414, insulin-like growth factor I, and Tudor domain-containing protein 7 (Table 2). These proteins are not only involved in the functions of maintaining cellular morphology, mechanical support, and load-bearing but also play roles in defense, protection, nutrition, and repair (45–47). Nevertheless, their biological functions after being absorbed by ticks are unknown.

## 5 Conclusion

Overall, tick hemolymph has a powerful transport function. Its transport function is largely dependent on its internal proteins. Our data indicated that 7 tick proteins and 13 host proteins were identified from hemolymph protein complexes by electrophoretic technique and the LC–MS/MS method. The five Vg family proteins and host serum albumin make up a large portion of hemolymph proteins. It is possible that these two types of proteins work together to participate in the transport of a variety of substances, such as fatty acids. This finding may offer fresh insights into the biology of ticks and, consequently, novel approaches to tick management.

## Data availability statement

The data presented in the study are deposited in the iProX repository, accession number PXD053899.

## Ethics statement

The animal study was approved by the Hunan Agricultural University Institutional Animal Care and Use Committee (No. 2021085). The study was conducted in accordance with the local legislation and institutional requirements.

## Author contributions

DL: Writing – original draft. LL: Writing – review & editing. Z-lL: Data curation, Writing – review & editing. YT: Data curation, Writing – review & editing. XG: Supervision, Writing – review & editing. T-yC: Writing – review & editing.

## Footnotes

^1^ https://maxquant.net/maxquant/

^2^ https://www.ncbi.nlm.nih.gov/bioproject/PRJNA756707/

^3^ https://www.ncbi.nlm.nih.gov/bioproject/?term=PRJNA600997

^4^ https://www.ncbi.nlm.nih.gov/bioproject/?term=GSE69721

^5^ https://www.ncbi.nlm.nih.gov/bioproject/?term=GSE67247

^6^ http://ibs.biocuckoo.org/
